# Parasitic Infection Surveillance in Mississippi Delta Children

**DOI:** 10.4269/ajtmh.20-0026

**Published:** 2020-06-22

**Authors:** Richard S. Bradbury, Irene Arguello, Meredith Lane, Gretchen Cooley, Sukwan Handali, Silvia D. Dimitrova, Fernanda S. Nascimento, Sam Jameson, Kathryn Hellmann, Michelle Tharp, Paul Byers, Susan P. Montgomery, Lisa Haynie, Brian Kirmse, Nils Pilotte, Steven A. Williams, Charlotte V. Hobbs

**Affiliations:** 1 Division of Parasitic Diseases and Malaria, Parasitic Diseases Branch, Center for Global Health, Centers for Disease Control and Prevention, Atlanta, Georgia;; 2 School of Health and Life Sciences, Federation University, Berwick Campus, Melbourne, Australia;; 3 Department of Pediatrics, Division of Infectious Diseases, Children’s of Mississippi, Jackson, Mississippi;; 4 Division of Genetics, Department of Pediatrics, University of Mississippi Medical Center, Jackson, Mississippi;; 5 The Delta Mercy Project, School of Nursing, University of Mississippi Medical Center, Jackson, Mississippi;; 6 Mississippi State Department of Health, Jackson, Mississippi;; 7 Department of Biological Sciences, Smith College, Northampton, Massachusetts;; 8 Program in Molecular and Cellular Biology, University of Massachusetts, Amherst, Massachusetts;; 9 Department of Microbiology, University of Mississippi Medical Center, Jackson, Mississippi

## Abstract

Some recent studies suggest ongoing transmission of parasitic diseases in the American South; however, surveys in Mississippi children are lacking. We enrolled 166 children (median age 8 years, range 4–13 years) from the Mississippi Delta region and carried out multi-parallel real-time polymerase chain reaction (PCR) for *Necator americanus*, *Ascaris lumbricoides*, and *Strongyloides stercoralis* on their stool samples. Dried blood spots were obtained for multiplex serology antibody detection. Of 166 children, all reported having flushable toilets, 11% had soil exposure, and 34% had a pet dog or cat. None had prior diagnosis or treatment of parasitic disease. Multi-parallel real-time PCRs were negative on the 89 stool DNA extracts available for testing. Dried blood spot testing of all 166 children determined the seroprevalence of IgG antibodies to *Toxocara* spp*.* (3.6%), *Cryptosporidium* (2.4%), *S. stercoralis*, *Fasciola hepatica*, and *Giardia duodenalis* (all 0%). In conclusion, parasitic infections and exposure were scarce in this population. Larger studies of at-risk populations are needed.

## INTRODUCTION

Hookworm and other soil-transmitted helminths (STHs) are diseases of poverty, which adversely affect the physical and cognitive development of children.^1^ In response to widespread hookworm STH infection in the Southeastern United States in the early twentieth century, the Rockefeller Sanitary Commission for the Eradication of Hookworm Disease was established in 1909. Over the following 5 years, the commission found that the prevalence of hookworm disease in Mississippi schoolchildren was 36.7%.^2^ Following the eradication efforts and with improved sanitation and economic development, it was subsequently assumed that hookworm, and other STHs were unlikely to be a continued problem in the American South.^1,3^ However, the last comprehensive STH surveillance study in the Southeast was in 1975,^4^ and STH cases continued to be reported to the Mississippi State Department of Health (MSDH) until the year 2000. The last report of hookworm to the MSDH was in 1992, when three cases were reported, as well as 11 cases of ascariasis and six of strongyloidiasis, although active reporting ceased in the preceding decade (B. Brackin, personal correspondence).

A 2017 study conducted in rural Alabama reported 19/55 and 4/55 stool samples positive by real-time polymerase chain reaction (PCR) for *Necator americanus* and *Strongyloides stercoralis*, respectively. This has highlighted the possibility of continued foci of STH infection in the Southeastern United States.^5^ We conducted a pilot study to estimate the prevalence of STH and other potentially endemic parasitic infections of schoolchildren in the Mississippi Delta region. The present study specifically targeted children in Sharkey County, Mississippi, where the median per capita income was $15,430 in 2016.^6^

## METHODS

### Survey of schoolchildren.

The survey of schoolchildren took place in Sharkey County, Mississippi, at a school-based health clinic run by the “Delta Mercy Project” (the University of Mississippi School of Nursing). At enrollment, children aged 2–18 years whose parent gave consent were eligible for enrollment; participating children older than 9 years provided assent. Basic history and physical examination were also collected/performed.

Within 1 year post-enrollment, on a rolling basis, and in conjunction with Delta Mercy Project health fair screenings/school clinic visits, risk factor data including whether subjects resided in homes with flushable toilets, had animals (dog/cat), soil exposures, and prior diagnosis or treatment of parasitic disease were collected. In addition, one fresh fecal specimen per child was collected for microscopy and real-time PCR, and dried blood spots (DBS) were collected for antibody testing, but not contemporaneously. Stool samples were stored at 4°C, transported, and processed in the laboratory within 24–72 hours post-collection.

### Laboratory analysis.

On receipt of fecal specimens in the laboratory at the University of Mississippi Medical Center (UMMC), two aliquots of 250 mg were frozen for DNA extraction and storage. DNA was extracted using the SurePrep Soil DNA Isolation Kit (Fisher Scientific, Fair Lawn, NJ), with bead beating using zirconium beads (Benchmark, Sayreville, NJ) for 3 minutes. Where sample volume was greater than 1 g direct microscopy was performed on fresh samples, as well as the saturated salt (specific gravity 1.2) centrifugal flotation.^9^

DNA extracts were stored at −80°C until shipment on dry ice to CDC for real-time PCR analysis. At the CDC, samples were initially tested for inhibition using a novel human cytochrome B gene real-time PCR (Supplemental Table 1). Non-inhibitory samples were then tested by multi-parallel real-time PCR for *N. americanus*, *Ascaris lumbricoides*, and *S. stercoralis*.^8,9^ A Ct value of ≤ 35 was considered a positive result. Plasmid DNA-positive controls^8^ were used for each real-time PCR, and a PCR-grade water-negative control was incorporated into each run.

Dried blood spots (DBS) from all schoolchildren were collected on Whatman 903 filter paper (Cardiff, United Kingdom) and tested for IgG antibodies to *Toxocara* spp.,^10^ *S. stercoralis*,^11^ *Fasciola hepatica*,^12^ *Cryptosporidium parvum*, and *Giardia duodenalis*^13^ using multiplex bead assays (MBAs) read via MAGPIX (Austin, TX) instruments to determine evidence of exposure to these diseases. All samples positive for *Toxocara* spp., *S. stercoralis*, or *F. hepatica* by multiplex serology were confirmed by Western blot. Full methods of MBA analysis are available in Supplemental Data.

### Data storage/statistics.

Data were logged in from case report forms and laboratory results into REDCap (https://www.project-redcap.org), and Microsoft Excel (2016, Microsoft Corporation, Redmond, WA) was used for all calculations.

### Ethics.

This study was approved by the UMMC Institutional Review Board; on ethical review, the CDC was determined to be non-engaged. Permission was obtained from the school principal, and parents or guardians of children enrolled provided written permission for participation for the infants enrolled in this study. Children aged 9 years and older provided assent. All samples sent to the CDC were de-identified. No drug was administered as part of this observational study, but schoolchildren received standard of care management for illness from the Delta Mercy Health Care Clinic and UMMC medical personnel.

## RESULTS

Overall, 166 children (median age 8 years, range 4–13 years) were enrolled in the study, including 75 males and 91 females. This study took place between July 2016 and August 2017.

No children reported prior parasitic disease diagnosis or treatment or living in a home with a non-flushable toilet. One-third (34%) of children reported having a pet cat or dog at home, whereas 11% reported soil exposure (walking barefoot or handling soil) (Table 1).

**Table 1:** Risk factors for soil-transmitted helminth and other parasitic infections as assessed by questionnaire of schoolchildren in Sharkey County, Mississippi, 2016 (*n* = 166)

Risk factor	Yes, *n* (%)
No flushable toilets in the home	0 (0)
Soil exposure	18 (11)
Pets (dog/cat)	56 (34)
Prior diagnosis or treatment of intestinal parasite	0 (0)

A total of 100 fecal samples were obtained. Only 16 fresh fecal samples from schoolchildren had sufficient volume to allow both DNA extraction and microscopy, and all of these microscopy results were negative. Eleven samples (11%) were negative by the inhibition/extraction control testing and were not further tested. The remaining 89 samples were tested by multi-parallel STH real-time PCR for *N. americanus*, *A. lumbricoides*, and *S. stercoralis* and found to be negative (Table 2).

**Table 2:** Results of multi-parallel real-time PCR for selected parasitic infections on fecal samples from schoolchildren in Sharkey county, Mississippi, 2016–2017 (*n* = 100)

Multi-parallel real-time PCR	Positive, *n* (%)
Human cytochrome B gene*	89 (89%)
*Necator americanus*	0 (0)
*Strongyloides stercoralis*	0 (0)
*Ascaris lumbricoides*	0 (0)

PCR = polymerase chain reaction.

* Extraction/inhibition control.

All 166 children enrolled provided a DBS sample for multiplex serology testing. None of the samples tested positive for antibodies reacting with the *S. stercoralis* Ss-NIE-1 antigen or the combined *G. duodenalis* VSP3 and VSP5 antigens. Antibodies reacting with both the *C. parvum* Cp17 antigen (range 558–10,823 MFI) and with *C. parvum* Cp23 (range 331–16,451 MFI) were detected in four of 166 samples (2.4%), which are required to determine a sample as positive. Antibodies to *Toxocara* spp. rTc-CTL-1 antigen (range 30–98 MFI) were detected in six of 166 samples (3.6%). Antibodies reacting with the *F. hepatica* rFh-SAP2 antigen (76 MFI) were detected in one of 166 samples (0.6%), but this sample tested negative in the *F. hepatica* confirmatory Western blot assay (Table 3).

**Table 3:** Seroprevalence rates for selected parasitic infections in dried blood spots collected from schoolchildren in Sharkey County, Mississippi, 2016–2017 (*n* = 166)

Multiplex serology	Positive, *n* (%)
*Toxocara* species	6 (3.6)
*Strongyloides stercoralis*	0 (0)
*Fasciola hepatica*	0 (0)*
*Cryptosporidium parvum*	4 (2.4)
*Giardia duodenalis*	0 (0)

* One sample positive by MAGPIX multiplex serology was found to be negative by the confirmatory Western blot test.

## DISCUSSION

We found a very low prevalence of parasitic diseases in children from this specific region of the Mississippi Delta. In this population, PCR and limited microscopy data suggest a lack of transmission of the important STH species in question: *N. americanus*, *A. lumbricoides*, and *S. stercoralis*.

Our risk factor data also suggested that most children enrolled did not have risk factors that would increase their likelihood of STH infection. These results may be subject to bias as certainly there is a stigma attached to the questions regarding soil exposure and the type of toilet in the house. Poverty is closely linked to a number of these infections,^4,14^ and the children enrolled came from a county in which the average income is below the poverty line cutoff for a household of four.^6^

The seroprevalence of *Toxocara* spp. in this school-based study is within the range of estimated prevalences in the age-groups of 6–11 (3.0%) and 12–19 (3.9%) years that were recently published in a larger national study.^15^ With respect to *Cryptosporidium*, the 2.4% prevalence in this small study of school-aged children is lower than in previous reports, although the comparison data are older and our study includes slightly younger children.^15^ The seroprevalence of *Giardia* was zero in this study, suggesting no recent exposure.

Our PCR and limited microscopy data suggest the absence of *N. americanus*, *A. lumbricoides*, and *S. stercoralis* infection in this population. It should be considered that Sharkey County had a historically low prevalence of *N. americanus* infection, with the Rockefeller Sanitary Commission reporting no more than 5.0% prevalence.^16^ The slowly permeable clay alluvium soils of Sharkey County are not generally conducive to hookworm development, which is best achieved in aerated sandy or loamy soil.^17–19^

Of note, because we concluded that our PCR results were negative and because PCR is not the current standard of care test used for diagnosis of STH infections, no children were referred for treatment. In the absence of signs and symptoms due to infection, the positive results for *Toxocara* and intestinal protozoa antibodies likely indicated exposure only and were not indication for treatment referral. Results for this study have been disseminated back to the community. All data (deidentified) have been communicated to the Mississippi Department of Health.

In comparison to a recent study performed in Lowndes County, Alabama, our results indicate negligible transmission of STHs and intestinal protozoa among children from Sharkey County, Mississippi. However, the possibility that isolated foci of STH infection remaining in marginalized communities that have poor sanitation and hygiene and poor access to health care, as observed in other developed countries such as Australia,^20^ cannot be excluded by the data presented here. Further investigation targeting counties specifically chosen for the aforementioned risk factors, as well as testing of additional age-groups, is warranted.

## Supplemental table

Supplemental materials
