# Supplementary material for: Parasitic Infection Surveillance in Mississippi Delta Children
Source: Am J Trop Med Hyg. 2020 Jun 22;103(3):1150–3. doi: 10.4269/ajtmh.20-0026 (PMC7470556; doi:10.4269/ajtmh.20-0026)
Supplement: Supplementary file 1 [file tpmd200026.SD1.doc]

**Supplementary data:** Development and validation of human cytochrome B gene real-time PCR assay

For the purpose of this study, real-time PCR based on cytochrome B gene was developed as an internal control test, to detect occurrence of real-time PCR inhibition. The reaction was validated using DNA extracts from human samples (blood, tissue and stool), stool samples from pigs and dogs were tested as negative controls and these did not show positive results. The PCR conditions were as follows; initial hold at 50°C for 2 minutes, followed by denaturation at 95°C 2 mins and then 40 cycles at 95°C for 15 secs and 59°C for 1 min.

| Forward primer | Hu-F | TCT TGC ACG AAA CGG GAT CA |
| --- | --- | --- |
| Reverse primer | Hu-R | CGA GGG CGT CTT TGA TTG TG |
| Probe | Hu-P | CTA GGA ATC ATC ACC TCC CAT TC - Hex |

**Supplementary table 1:** Primers and probes for human cytochrome B gene real-time PCR used for control of DNA extraction and detection of inhibition purposes in this study

*Full methods of multiplex bead assay serological analyses*

For *Toxocara* spp., *S. stercoralis*, and *F. hepatica* antibody detection, dried blood spots were punched into 3 mm diameter using a handheld hole punch. Four 3 mm dried blood spots from each sample were placed in 250 µL of elution buffer (PBS + 0.3% Tween-20 + 0.1% sodium azide (NaN_3_) and allowed to elute overnight at 4° C. Afterward, the elution was further diluted 1:2 with the same elution buffer containing 10 grams of dry skim milk to give a final dilution of 1:100. The eluate was exposed to antigen-coupled beads (either rTc-CTL-1 antigen for detection of antibody against -*Toxocara* spp.,^9^ rSs-NIE-1 for *S. stercoralis*,[^12^](#_ENREF_12) or rFh-SAP2 for *F. hepatica*^11^) diluted in the same diluent for 30 minutes, shaking at room temperature. Bound antigen-specific IgG or IgG4 (for *S. stercoralis*) were detected on the coupled beads as previously described.^10^ Between steps, the magnetic beads were washed three times with 0.05% Tween 20 in PBS, using a BioTek Plate washer (BioTek Instruments, Winooski, VT). A MAGPIX reader with xPONENT ® software (Luminex) calculated the median fluorescence intensity from each bead classification from each well. Background fluorescence from a blank with no dried blood spot was subtracted (MFI-bg, reported as MFI for short) and used as data. Receiver operating characteristic (ROC) curve analysis was used to define cutoffs as previously described with 8 MFI for *S. stercoralis*,[^12^](#_ENREF_12) 23.1 MFI for *Toxocara* spp.,[^11^](#_ENREF_11) and 27.8 MFI for *F. hepatica*[*^13^*](#_ENREF_13) being used in the current study.

Antibodies against *C. parvum* Cp17 and Cp23 and *G. duodenalis* VSP3 and VSP5 were measured using a different format of MBA. One 3 mm circular punch was incubated overnight at 4° C in 500 µL Buffer B (1x PBS, 0.5% polyvinyl alcohol, 0.8% polyvinylpyrrolidone, 0.5% casein [all Sigma, Burlington MA], 0.3% Tween-20, 0.02% sodium azide) containing 3 µg/mL of *Escherichia coli* extract. Antigen-specific IgG was detected by incubating specimens in duplicate with beads and then detecting with 50 ng per well of monoclonal mouse anti-human IgG and 20 ng per well of IgG4 (both Southern Biotech, Birmingham AL) and 250 ng per well of streptavidin-linked R-phycoerythrin reporter (Invitrogen, Waltham MA), as described previously.[^22^](#_ENREF_22) Antibody binding was detected using MAGPIX instrument and reported as median fluorescence intensity minus background from wells containing only Buffer B with the *E. coli* extract to generate the final MFI-bg values (reported as MFI in short). Specimens having a coefficient of variation of >15% between the MFI of duplicate wells for any bead region were repeated. Cutoffs for Cp17 (115 MFI) and Cp23 (285 MFI) were extrapolated from previous cutoffs determined by ROC curve analysis of sera classified as positive or negative to *Cryptosporidium* oocyst lysate in Western blot. A panel of 86 adults with no known exposure to *Giardia* was used to calculate the mean plus three standard deviations to determine cutoffs for VSP3 (105 MFI) and VSP5 (265 MFI). A sample would need to be positive for both antigens (Cp23 and Cp17 or VSP3 and VSP5) to be considered positive for exposure to *Cryptosporidium* or *Giardia*.
